# Advancing Health Equity Through Primary Care: Protocol for the Spread, Scale, and Multimethod Developmental Evaluation of the Deep End Canada Network

**DOI:** 10.2196/75732

**Published:** 2025-10-10

**Authors:** Joseph J O'Rourke, Mélanie Ann Smithman, Isabelle Fortuna, Ellah San Antonio, Archna Gupta, Leanne Kosowan, Andrew D Pinto

**Affiliations:** 1 Upstream Lab, MAP Centre for Urban Health Solutions Li Ka Shing Knowledge Institute, St. Michael's Hospital Unity Health Toronto Toronto, ON Canada; 2 Department of Family and Community Medicine, St. Michael's Hospital Unity Health Toronto Toronto, ON Canada; 3 Department of Family and Community Medicine University of Toronto Toronto, ON Canada; 4 Department of Family Medicine Rady Faculty of Health Sciences University of Manitoba Winnipeg, MB Canada; 5 Dalla Lana School of Public Health University of Toronto Toronto, ON Canada

**Keywords:** spread, scale, primary health care, innovation, developmental evaluation, demographic data, social needs, health equity

## Abstract

**Background:**

The social determinants of health are “the conditions in which people are born, grow, live, work and age,” such as housing, employment, and race. Canadian primary health care organizations are increasingly looking for ways to systematically and routinely collect demographic and social needs data from patients to increase appropriate and responsive care by attending to the social determinants of health. The SPARK (screening for poverty and related determinants to improve knowledge of and links to local resources) tool is a standardized tool for use in primary health care settings that was developed, pilot-tested, refined, and validated in primary health care clinics across Canada. General Practitioners at the Deep End is a network of primary health care general practitioners working in the 100 most socioeconomically deprived areas in Scotland with the goal of connecting practitioners and advocating for better training, policies, and resources. Primary Health Care at the Deep End Canada or Soins primaires en milieux défavorisés Canada (Deep End Canada) was launched in June 2024 to offer guidance and resources to the growing number of primary health care teams interested in collecting and using demographic and social needs data.

**Objective:**

Our primary objective is to spread the sustainable implementation of the SPARK tool to systematically and routinely collect and use demographic and social needs data to respond to the social determinants of health in 20 to 25 primary health care organizations across Canada through the creation of Deep End Canada.

**Methods:**

Deep End Canada is a network of primary health care teams, including health professionals, researchers, and patient partners, working in areas with high rates of poverty. This project will use a multimethod developmental evaluation approach guided by the reach, effectiveness, adoption, implementation, and maintenance framework. Web-based surveys will capture the reach and adoption of demographic and social needs data collection, and network activities will be evaluated using qualitative analysis of focus groups, interviews, and meetings.

**Results:**

The study was funded in February 2022. Recruitment to the network commenced in June 2024 and included 10 organizations as of submission of the manuscript. Web-based survey data collection commenced in September 2024. Implementation of the network will be assessed from June 2024 to December 2025, with expected findings available in June 2026 and published in the fall of 2026.

**Conclusions:**

Efforts to systematically and routinely collect and use demographic and social needs data and address structural issues in Canadian primary health care have grown significantly in the last 10 years. This increased commitment involves using data to develop evidence-based interventions that can reduce health inequities and address local community needs at the individual, organizational, and policy levels. This study will demonstrate how the Deep End Canada network contributes to achieving those aims by fostering collaboration among practitioners to promote health equity and tackle the structural and social determinants of health.

**International Registered Report Identifier (IRRID):**

DERR1-10.2196/75732

## Introduction

### Background

The social determinants of health are “the conditions in which people are born, grow, live, work and age” [1], such as income, educational level, housing, employment, social support, food security, race, and gender. These determinants are pivotal in shaping health across the life span and contribute to health inequities. There is a growing recognition that health care should address the social determinants of health to achieve better and more equitable health outcomes and meet health system goals such as the quadruple aim [2]. Major health organizations, including the World Health Organization [3], British Medical Association [4], Canadian Medical Association [5], College of Family Physicians of Canada [6], and Registered Nurses’ Association of Ontario [7], have called on health systems to play a greater role in tackling the social determinants of health. However, a recent review of the literature on addressing social determinants in health care settings found that evidence supporting such interventions remains limited [8]. Addressing the social determinants of health directly and going “upstream” of illness and disease remains uncommon in health systems, including in Canada [9].

In Canada, primary health care is typically the first contact that a patient has with the health care system and offers a strong opportunity to address patients’ social needs. Primary health care is community based; focused on long-term patient relationships; and comprehensive, with a focus on systematically addressing broader social, economic, environmental, and individual determinants of health [10]. Indeed, the last decade has seen a major advancement in the involvement of primary health care teams in direct interventions on the social determinants of health [6,11-13]. Initiatives encompass clinical tools, educational sessions, and clinical guidelines for medical trainees and physicians, as well as medical-legal partnerships addressing housing, employment issues, immigration, citizenship, and social benefits [14-18]. A central priority of primary health care is commitment to health equity, the ability of everyone to achieve their full health potential without disadvantage regardless of their social position [19].

Despite advances, primary health care organizations and primary care providers face barriers to identifying and addressing social needs, including a lack of time, a lack of expertise, limited knowledge of community resources, education and training focused on a biomedical understanding of health and disease, and funding directed to “downstream” and acute health concerns [20-22]. Moreover, there is a lack of individual-level patient data necessary to accurately screen patients’ needs and develop targeted interventions [8,9,23]. Demographic and social needs data collected through routine and systematic screening paired with interventions to meet identified social needs could help further address the social determinants of health in primary health care [23-27]. Among health organizations that collect demographic and social needs data, many do not link the data to their electronic medical records (EMRs), and when they do, the quality is demonstrably variable [24,28]. Robust demographic and social needs data linked to EMRs could support quality improvement, the development of new care pathways at the organizational level, and planning and resource allocation at the system level [29,30]; bolster public health [22]; and advance research on health inequities [9,31,32].

While several screening tools to collect individual-level demographic and social needs data have been piloted [33-36], most have been tested in a specific setting, mainly in the United States, and little research has focused on how these tools can be effectively implemented, spread, and scaled in diverse primary health care practices. The SPARK (screening for poverty and related determinants to improve knowledge of and links to local resources) tool is a standardized tool for use in primary health care settings developed, pilot-tested, refined, and validated in primary health care clinics across Canada over the past decade building on health equity questions developed in 2012 [37-41]. The SPARK tool includes 17 questions that assess demographics (language, immigration status, Indigenous identity, race, disability status, gender identity, and sexual orientation) and social needs (educational level, income, food security, medication access, housing status, transportation, phone and internet access, cost of utilities, social isolation, and precarious employment) plus 2 optional questions (ethnicity and religion). The spread and scale of demographic and social needs data collection requires an organizational commitment to collecting and using such data to inform care provision that can respond to the social determinants of health and reduce health inequities.

General Practitioners at the Deep End is a network of primary health care general practitioners (GPs) working in the 100 most socioeconomically deprived populations in Scotland with the goal of facilitating connections between practitioners working in deprived regions [42]. It uses Julian Tudor-Hart’s swimming pool analogy for the inverse care law to highlight how patients living in the most deprived regions (in the “deep end”) often have the highest health care needs but the lowest availability and quality of health care resources [43]. Since its inception in 2009, General Practitioners at the Deep End has grown beyond a peer support network to include 20 networks in 9 countries that advocate for better training, policies, and resources for their communities and patients. Deep End projects have propagated across the United Kingdom and the world, with networks in Ireland, Australia, and Japan, among other locations [44,45]. While some follow the original model of inviting practices from regions with the highest deprivation scores, others are open to all GPs with an interest in addressing health inequities. Deep End activities can include regular meetings for physician networking and support, policy and advocacy initiatives, and fellowship programs to support physicians working in areas of socioeconomic deprivation [44-46].

**Figure 1:**
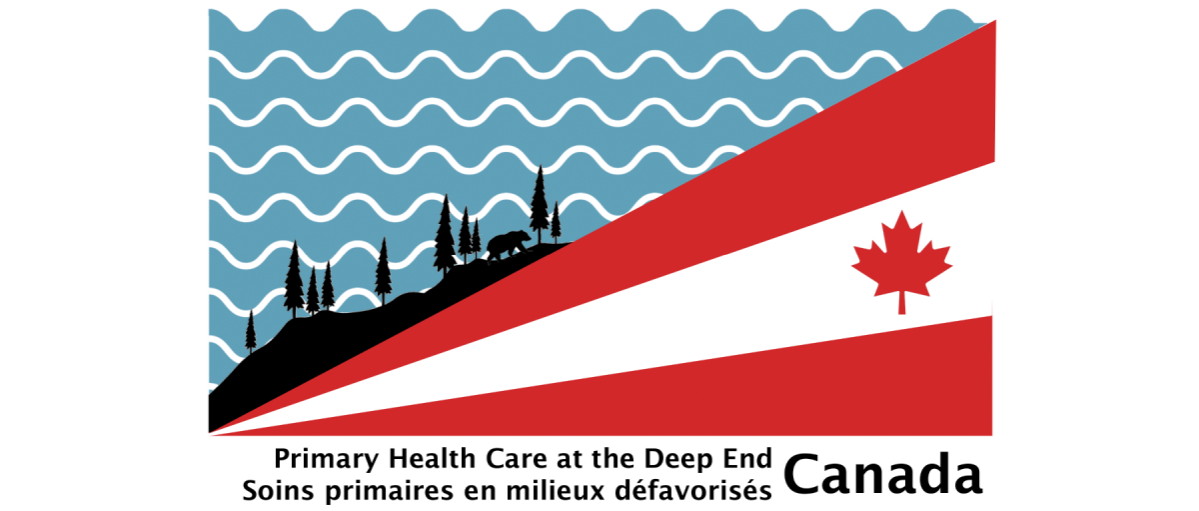
Primary Health Care at the Deep End Canada / Soins primaires en milieux défavorisés Canada logo.

Primary Health Care at the Deep End Canada / Soins primaires en milieux défavorisés Canada (Deep End Canada) was launched in June 2024 (Figure 1), joining the Deep End international network [47]. The network builds on growing interest among Canadian primary health care in collecting and using demographic and social needs data to address social determinants of health by offering resources and guidance. Deep End Canada offers clinics over 25 resources to guide demographic and social needs data collection and use (eg, a copy of the SPARK tool in English and French and patient and staff informational materials), regular coaching meetings with Deep End Canada coordinators, and the opportunity to meet with other clinics across Canada. Deep End Canada’s mission is to advocate for addressing health inequities in primary health care at the individual, organizational, and policy levels through the collection and use of demographic and social needs data and develop, share, and collaborate on clinic-level initiatives and projects. The network consists of primary health care teams, including health professionals, researchers, patient partners, and decision-makers, working with patients who may face social or economic disadvantages.

### Objectives

By establishing Primary Health Care at the Deep End Canada, our primary objective is to assist 20 to 25 primary health care clinics or organizations across Canada attend to their patients’ social determinants of health through the sustainable implementation of the SPARK tool to systematically and routinely collect and use demographic and social needs data. Our secondary objective is to facilitate community engagement in the collection and use of demographic and social needs data to identify and address inequities.

### Research Questions

The research questions (RQs) for this study are as follows:

1. What is the reach, effectiveness, adoption, implementation, and maintenance of the SPARK tool and other demographic and social needs data collection efforts in primary health care settings across Canada?
2. What factors influence the use of social needs data to advance individual-, organizational-, and policy-level interventions addressing social determinants of health for clinics part of Deep End Canada?
3. How does Deep End Canada facilitate community and key actor engagement between and beyond members? How do these activities support the collection and use of demographic and social needs data?

## Methods

### Overview

Primary Health Care at the Deep End Canada is a network of primary health care teams, including health professionals, researchers, patient partners, and decision-makers, working in underresourced areas of Canada with patients who may face social or economic disadvantages [48]. Their central goal is the same: to advocate for addressing health inequities in primary health care at the individual, organizational, and policy levels and develop, inform, and share strategies that can support data collection, data use, and related local health equity initiatives. Deep End Canada members engage with the network in 3 ways. First, they attend quarterly network meetings to share opportunities for collaboration, challenges, and strategies related to health equity with other clinics across Canada. Second, they participate in regular implementation coaching sessions to support their use of the SPARK tool, analysis of demographic and social needs data, and the use of demographic and social needs data to develop health equity interventions. Third, they receive resources and materials to support demographic and social needs data collection in primary health care [37]. Network activities may include, for example, summarizing evidence of primary health care interventions to tackle social determinants (eg, environmental scans of best practices), supporting local spread and scale efforts (eg, helping clinics expand demographic and social needs data collection to other local sites and advocating for regional or provincial expansion), and responding to new opportunities or policies (eg, supporting demographic and social needs data collection integration into new EMR systems).

### Overall Design and Approach

This project aims to gain a better understanding of what factors can support the development and maintenance of a network of primary health care clinics focused on the systematic and routine collection and use of demographic and social needs data, with an overarching aim of addressing patients’ social determinants of health at the individual, clinic, organizational, community, and policy levels. This project will use a multimethod developmental evaluation approach guided by the reach, effectiveness, adoption, implementation, and maintenance (RE-AIM) framework [49-53], as described in Table 1.

**Table 1:** Developmental evaluation guided by the reach, effectiveness, adoption, implementation, and maintenance (RE-AIM) framework.

RE-AIM outcome [54,55]	Key pragmatic priorities to consider	Answers
Reach	Who is intended to benefit and who actually participates or is exposed to the “project” or policy?	Primary health care settings focused on tackling social determinants of health and patients with social needs
Effectiveness	What is the most important benefit one is trying to achieve and what is the likelihood of negative outcomes?	Adoption and implementation of routine and systematic collection and use of demographic and social needs data at the individual level and planning for use at the clinic level
Adoption	Where is the project or policy applied? Who applies it?	Primary health care settings at the clinic or organizational level Primary care providers, clerical staff, operations staff, clinic leadership, and quality improvement teams
Implementation	How consistently is the project or policy delivered? How will it be adapted? How much will it cost? Why will the results come about?	Regular coalition meetings as determined by the coalition and demographic and social needs data collection as determined by the clinic Web-based (eg, manual or software EMR^a^ integration, EMR survey, or public URL survey) or on paper Cost of implementation depends on the clinic setting and implementation strategy Results will come about as per data collection outlined in this protocol
Maintenance	When will the project become operational, how long will it be sustained (setting level), and how long are the results sustained (individual level)?	June 2024 until June 2026 Sustainment at the individual and setting level will vary by clinic due to local factors (eg, clinic size, number and availability of staff and primary care providers)

^a^EMR: electronic medical record.

### Reach and Adoption: Pan-Canadian SPARK Survey (RQ 1)

The study team will use a combination of the SPARK reach and adoption survey and follow-up survey to learn about the reach and adoption of the SPARK tool and demographic and social needs data collection efforts across Canada. We will use a short electronic survey to collect background and contextual information from each primary health care clinic interested in using the SPARK tool or collecting demographic and social needs information (Multimedia Appendix 1). Information collected will include, for example, name and email address, location, type of organization, role, size of clinic or organization, familiarity with demographic and social needs questions, and intended or actual adoption of the SPARK tool. A follow-up survey at 6 months will include an assessment of perceptions and actions related to demographic and social needs data collection and participation in Deep End Canada after a 6-month period. The spread and scale of primary health care innovations has been previously evaluated using surveys at multiple time points [56]. Furthermore, clinics not already part of Deep End Canada may develop an interest in joining the network after they complete the survey. Draft copies of the 2 surveys are available in Multimedia Appendix 2.

### Effectiveness, Implementation, and Maintenance: Deep End Canada Activities

The effectiveness, implementation, and maintenance components of the RE-AIM framework will be used to evaluate other potential Deep End Canada activities identified by members. Deep End Canada members will generate potential ideas for implementing and evaluating their local efforts to collect demographic and social needs data and use them to support care responsive to the social determinants of health, as well as other local, regional, provincial, or national efforts to advance health equity at the individual, organizational, and policy levels. These discussions will support the development of measures that Deep End Canada can apply to evaluate both the effectiveness, implementation, and maintenance of its network and the network’s ability to support clinic activities. Furthermore, implementation and evaluation ideas will develop and grow alongside changes and growth in Deep End Canada membership, which may include clinics in different urban and rural areas, clinics focused on health care for different priority groups (eg, homelessness, addiction medicine, newcomers, and refugees), or Indigenous health clinics.

Demographic and social needs data collection will be one of several activities that Deep End Canada members may implement as part of their work on addressing social determinants of health. Many patients may not feel comfortable sharing personal demographic or social needs information or feel that it is relevant for their primary care providers. Deep End Canada facilitators will make relevant resources available to help make patients feel comfortable, including key messages for clerical staff and primary care providers to emphasize why the clinic is collecting demographic and social needs data, that the tool is voluntary, that responses are only visible to the health care team and protected by law like all health information, and that researchers will not be able to identify patients. As facilitators of Deep End Canada, we also have resources that emphasize the importance of community engagement and data governance to facilitate appropriate collection and use of data, especially if organizations plan to ask questions regarding Indigenous identity. Resources may be shared directly with clinics during or following coaching sessions or widely distributed to the network depending on interest and need.

Other activities of Deep End Canada could include system-level efforts to support demographic and social needs data sharing between organizations or collective policy advocacy for advancing health equity. For example, Deep End Canada members may choose to engage in initiatives such as creating a pan-Canadian set of demographic and social needs data; identify opportunities for collective policy advocacy; or facilitate deliberative dialogues with policymakers, patients, and other key stakeholders to identify system-level opportunities for advancing health equity.

### Deep End Canada Evaluation: Meeting Minutes and Tracking Sheet (RQs 2 and 3)

As a starting point, the developmental evaluation of Deep End Canada activities will first involve analyzing quarterly meeting minutes using the RE-AIM framework to share back to all network members at Deep End Canada meetings and inform discussions about potential activities, lessons for other clinics, and ongoing challenges at specific clinics or for the entire network. Second, we will use a tracking sheet to evaluate engagement with prospective and current Deep End Canada members after implementation coaching sessions and email communications. This sheet will also help us understand the implementation and effectiveness of the network in helping clinics set and achieve goals related to demographic and social needs data collection and use and health equity interventions (drawing on quality improvement methodology such as cycles of planning, doing, checking, and acting).

### Deep End Canada Evaluation: Focus Groups and Interviews (RQs 2 and 3)

Throughout the evaluation, we will engage Deep End Canada members to identify indicators of the effectiveness, implementation, and maintenance of their health equity activities, focus groups, or interviews. A draft copy of the focus group and interview guide is available in Multimedia Appendix 3. The study team will use focus groups and interviews with staff from various Deep End Canada member clinics to collect information on participants’ involvement in data-driven local solutions (eg, demographic and social needs data collection), community and key actor engagement (eg, involvement with Deep End Canada members and local communities), and social action and advocacy (eg, support for pan-Canadian campaigns related to primary health care). Focus groups were chosen as they offer the opportunity for people who share similar experiences and identities (eg, as primary health care staff) to discuss their experiences and opinions and learn from each other in a supportive, nonjudgmental, and neutral setting. Focus groups and interviews will last 45 to 90 minutes.

### Recruitment

Initial recruitment to Deep End Canada will involve self-selection of clinics that operate in underresourced areas of Canada with patients who may face social or economic disadvantages and are committed to tackling social issues to improve the health of their patients and communities. Survey participant recruitment will involve convenience sampling of staff at primary health care clinics in Canada who contact the authors for information about the SPARK tool or those who contact Deep End Canada or access the Deep End Canada website pages to download SPARK tool–specific implementation resources [57]. We are sharing information about the Deep End Canada website with representatives from primary health care teams who contact the coauthors about the SPARK tool given over a decade of published work on demographic and social needs data collection in primary health care [27,30,37-41], public health [22], and hospital settings [31,32]. We will use purposive sampling to contact clinics and organizations across Canada engaged in demographic and social needs data collection efforts either directly using publicly available contact information on their websites (or contact information provided when they contact us) or through relevant listserves and mailing lists (eg, the Canadian Institute for Health Information mailing list). In addition, clinics may be referred by other clinics involved (snowball sampling).

### Sample Size Rationale

The total sample size is 250 organizations. For the SPARK reach and adoption survey, the sample size (n=200) is based on an estimate of how many organizations across Canada expressed interest in using the SPARK tool previously and those we anticipate would be interested in learning more about it. For the meeting minutes, tracking sheet, focus groups, and interviews, the sample size (n=50 participants) is based on data from 2 to 4 participants from each of the 13 Deep End Canada member organizations at the time of writing.

### Inclusion and Exclusion Criteria

Survey participants will include staff from health clinics that indicate an interest in pursuing and supporting demographic and social needs data collection or health equity interventions in primary health care settings in Canada when contacting us. We will exclude participants who are under the age of 18 years.

The meeting minutes, tracking sheet, focus group, and interview participants will include Deep End Canada members at the time of recruitment (which involves meeting with the coordinating team and attending a network meeting at least once). At the time of writing, Deep End Canada has 13 member organizations representing over 25 primary health care clinics in 8 provinces (British Columbia, Alberta, Saskatchewan, Ontario, Quebec, Prince Edward Island, Nova Scotia, and Newfoundland and Labrador). Organizations include family health teams, university health centers, community health centers, primary care networks, community pharmacies, research teams, and clinical trial teams. Members include primary health care professionals, researchers, patient partners, and administrators supporting patients who may face social or economic disadvantages.

### Analysis

We will calculate descriptive statistics (frequencies and percentages and means and SDs) to summarize clinic characteristics from the reach and adoption surveys, such as geographic representation, types of clinics represented, or types of professions represented. The initial survey and optional follow-up survey will be linked to examine changes in perceptions and actions related to demographic and social needs data collection over 6 to 12 months. Surveys will be designed so that identifying information about the clinic is kept separately from the responses; however, a code will be embedded in the surveys to allow researchers to link the responses for analyses. We will analyze the differences between respondents who participate in one or more Deep End Canada network meetings or coaching sessions and those who do not. We may also examine how level of engagement (eg, regularly attending network meetings and coaching sessions vs engaging with Deep End Canada a single time) impacts responses.

We will transcribe interviews and focus groups verbatim. We will apply content analysis to the qualitative data, including interviews, focus group discussions, survey responses, or meeting minutes [56]. Using an initial codebook informed by the RQs (eg, RE-AIM framework, use of social needs data, and community engagement), we will analyze transcripts deductively and inductively. We will iteratively refine this codebook using the RE-AIM framework and other relevant activities of Deep End Canada members (eg, experiences designing and implementing health equity interventions or engaging in advocacy).

As a developmental evaluation, combining qualitative and survey data will provide a richer understanding of implementation outcomes and an in-depth understanding of context for and barriers to and facilitators of the implementation and maintenance of the SPARK tool or initiatives to respond to social determinants of health in primary health care, including system-level advocacy. This approach will also enable us to better understand participants’ perspectives of participating in a pan-Canadian network focused on health equity.

The sources of data collected in this study will help answer the overarching RQs and be combined to provide insights on the role of Deep End Canada in Canadian primary health care. First, survey data will allow us to understand the role of demographic and social needs data collection in building a foundation of health data and provide concrete examples of how clinics plan to or currently draw on such data when designing health equity interventions at the individual, clinic, organizational, or policy levels. Second, focus group and interview data will provide insights into how various activities broadly focused on reducing health inequities can be integrated and shared across a pan-Canadian network to support similar activities in other contexts. Finally, qualitative data, analyzed together with survey data, meeting minutes, and the tracking sheet as part of this overall study, will demonstrate how Deep End Canada serves as a basis not only for discussing specific activities such as demographic and social needs data collection and use but also for collaboration and mutual support across jurisdictions to identify and address system- and policy-level challenges within primary health care.

### Advisory Group

Our advisory group will include 4 to 6 patient partners from multiple provinces who have experience receiving primary health care in Canada or were involved in various phases of the SPARK study and development of the SPARK tool. Their role will include, for example, providing input on Deep End Canada member activities, participating in quarterly meetings, reviewing evaluation feedback to clinics, reviewing proposed initiatives to respond to social determinants of health from a patient perspective, interpreting findings, and helping develop recommendations for advancing the mission of Deep End Canada. This could include discussing the design and wording of patient-facing materials and ensuring the clarity of the network’s mission.

### Ethical Considerations

We will obtain informed consent and provide the option to opt out of the study for all study participants. All interviews, focus groups, and surveys will be deidentified before being aggregated for analysis. No participants will be identifiable when the findings are published. Focus group and interview participants will be offered a CAD $30 (US $21.71) honorarium for their time. The first survey was approved by the Unity Health Toronto Research Ethics Board (REB) on July 2, 2024 (24-094). At the time of writing, a REB amendment is under review to include the other data collection activities described in this protocol in the REB approval (follow-up SPARK reach and adoption survey, Deep End Canada evaluation activities, and focus groups and interviews).

## Results

**Figure 2:**
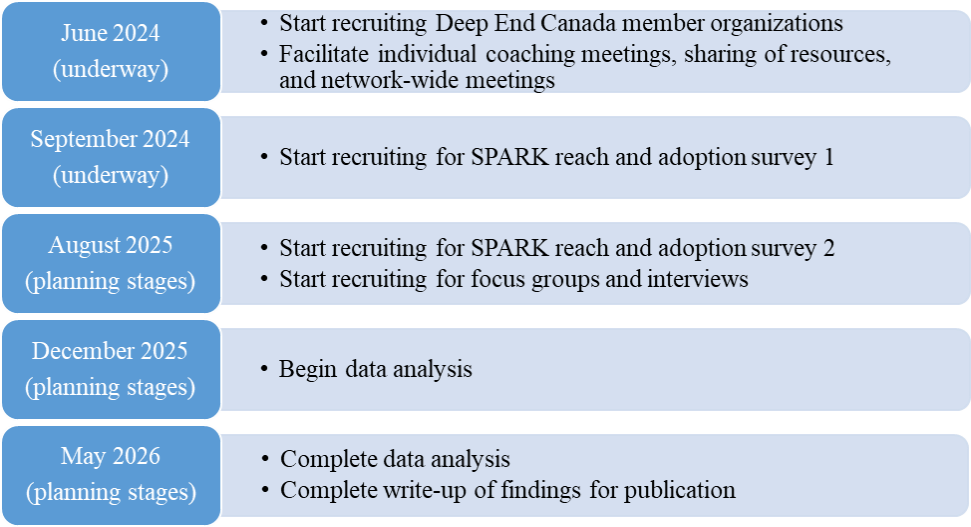
Schematic of project workflow. SPARK: screening for poverty and related determinants to improve knowledge of and links to local resources.

Recruitment to the network commenced in June 2024 and included 10 organizations as of submission of the manuscript. Data collection using a web-based survey commenced in September 2024. Implementation of the network will be assessed from June 2024 to December 2025, with expected findings available in June 2026 and published in the fall of 2026 (Figure 2).

## Discussion

### Anticipated Principal Findings

The anticipated findings of this study will include a cross-sectional view of the reach, effectiveness, adoption, implementation, and sustainability of the collection and use of demographic and social needs data across Canada (using the SPARK tool and other tools), highlighting the role of Deep End Canada in supporting and expanding such work to promote equitable health care. Other findings will include factors that influence the use of such data by members of Deep End Canada to advance individual-, organizational-, and policy-level interventions and community and key actor engagement to address social determinants of health.

### Comparison With Previous Work

Previous research on the development of Deep End networks provide peer support to members and have established policy and advocacy initiatives, and fellowship programs to support GPs working in areas of socioeconomic deprivation [44,45]. However, no Deep End networks have made an explicit commitment to building the capacity of primary health care to systematically and routinely collect and use demographic and social needs information alongside other health equity interventions. Deep End Canada will help build such capacity while also facilitating other activities associated with Deep End networks internationally, such as supporting the primary health care workforce and promoting education, advocacy, and research [45,58].

### Strengths and Limitations

The strength of this study is the use of an established international model for building collective identity and facilitating opportunities for social action in primary health care to improve patient care and health outcomes, especially for those experiencing poverty and who face social or economic disadvantages. This study will provide a realistic view of how a network of primary health care clinics committed to a common mission will facilitate the implementation and sustainability of health equity interventions, including the collection and use of demographic and social needs data. The focus of our study, in addition to describing an effort to expand demographic and social needs data collection across Canadian primary health care, is to generate “more uncertain answers to broader, more complex questions” [59]—we hope to provide a space for members to collaboratively discuss topics and develop interventions related to health equity that align with their interests and needs while acknowledging challenges associated with supporting patients experiencing poverty-related hardships and providing mutual support [43].

Limitations of this study include potential discrepancies between the goals of individual members and their vision for the network (eg, discussing ideas to address the social determinants of health of their patients) compared to the aims of the developmental evaluation (eg, reach of the SPARK tool and understanding the influence of the network on health equity interventions). For example, if members feel an imbalance where they are unable to address their interests or gain something from the network due to a greater emphasis on a particular topic or on collecting data to inform the evaluation (eg, structuring meeting agendas to discuss the SPARK tool), they may opt to leave the network and seek support elsewhere. Such challenges may preclude a full understanding of the network’s potential influence on members who may have joined for reasons other than collecting and using demographic and social needs data. Second, our approach to recruitment (ie, self-selection) may limit the involvement of clinics in rural settings or those with limited capacity to participate in activities that do not involve addressing the day-to-day clinical needs of patients or that do not know about the network as we primarily engage clinics actively seeking support.

### Future Directions

Future directions for Deep End Canada beyond helping build a foundation of demographic and social needs data from which to address social determinants of health across Canadian primary health care should include initiatives that address national issues in primary health care. Aligned with the workforce, education, advocacy, and research framework and adopted by several Deep End networks internationally [43,45,48], one priority that Deep End Canada could support is the national adoption of the OurCare standard, which represents “the collective aspirations of all people in Canada for a more sustainable, high-quality and equitable” primary health care system [60,61]. Future research could involve studying the factors that facilitate the implementation of the standard and how implementing it potentially reduces burnout in the primary health care workforce. Other future directions could include advocating for policies that reflect the standard and address issues in primary care and educational activities to share lessons from other networks internationally to support Deep End Canada and related initiatives [62,63]. Deep End Canada will continue to facilitate meetings for members to shape the network’s direction, with a focus on reducing health inequities [43].

### Policy Implications

Policy implications include the potential for Deep End Canada to provide ongoing mutual support for primary health care professionals supporting patients experiencing hardships related to poverty, with potential financial or human resources support from professional bodies such as the College of Family Physicians of Canada or the Canadian Medical Association. Evidence generated from this study could also lead policymakers to seek opportunities for encouraging the systematic and routine collection of demographic and social needs data, as was done for public health units in Ontario as part of the provincial COVID-19 response [22]. Finally, evidence generated from this study could offer insights into ways to conduct research, education, and patient and community engagement with primary health care clinics that are closely supporting patients experiencing poverty and other social needs in other contexts.

### Conclusions

Efforts to implement systematic and routine demographic and social needs data collection and use and address other structural issues in Canadian primary health care have grown significantly in the last 10 years. Researchers developed the Primary Health Care at the Deep End Canada national network of primary health care teams to not only collect but also use such data to develop evidence-based health equity interventions that aim to reduce health inequity and address needs within the local community at the individual, organizational, and policy levels. This study will demonstrate how the Deep End Canada network contributes to achieving those aims, drawing on national survey and qualitative data. Deep End Canada is based on an established international model born of the collective organizing of GPs in Scotland to build social connections to offer mutual support and collaborate to systematically address the health impacts of poverty on their patients and communities. It is in this spirit that the Deep End Canada network seeks to foster Canada-wide collaboration among multidisciplinary practitioners to promote health equity and tackle the structural and social determinants of health.

## Appendix

Multimedia Appendix 1 The SPARK (screening for poverty and related determinants to improve knowledge of and links to local resources) tool.

Multimedia Appendix 2 SPARK reach and adoption survey 1 and 2.

Multimedia Appendix 3 Draft focus group and interview guide.

## Abbreviations

EMR: electronic medical record
GP: general practitioner
RE-AIM: reach, effectiveness, adoption, implementation, and maintenance
REB: Research Ethics Board
RQ: research question
SPARK: screening for poverty and related determinants to improve knowledge of and links to local resources

## Data Availability

The datasets generated or analyzed during this study (where not already included in the published article with the study findings) will be available from the corresponding author on reasonable request.

## References

[1] (2008). Closing the gap in a generation: health equity through action on the social determinants of health. World Health Organization.

[2] Bodenheimer T, Sinsky C (2014). From triple to quadruple aim: care of the patient requires care of the provider. Ann Fam Med.

[3] Social determinants of health. World Health Organization.

[4] Marmot M Social determinants of health - what doctors can do. British Medical Association.

[5] Canadian Medical Association (2013). Physicians and health equity: opportunities in practice. The National Collaborating Centre for Determinants of Health.

[6] (2015). Best advice guide: social determinants of health. The College of Family Physicians of Canada.

[7] (2011). Creating vibrant communities: RNAO’s challenge to Ontario’s political parties. Registered Nurses' Association of Ontario.

[8] Andermann A, CLEAR Collaboration (2016). Taking action on the social determinants of health in clinical practice: a framework for health professionals. CMAJ.

[9] Pinto AD, Bloch G (2017). Framework for building primary care capacity to address the social determinants of health. Can Fam Physician.

[10] Gupta A, Gray CS, Landes M, Sridharan S, Bhattacharyya O (2021). Family medicine: an evolving field around the world. Can Fam Physician.

[11] Goel R, Buchman S, Meili R, Woollard R (2016). Social accountability at the micro level: one patient at a time. Can Fam Physician.

[12] Woollard R, Buchman S, Meili R, Strasser R, Alexander I, Goel R (2016). Social accountability at the meso level: into the community. Can Fam Physician.

[13] Meili R, Buchman S, Goel R, Woollard R (2016). Social accountability at the macro level: framing the big picture. Can Fam Physician.

[14] Raza D, Bloch G, Sonia TK, Kulie Office interventions for poverty. Health Providers Against Poverty.

[15] Bloch G, Etches V, Gardner C, Pellizzari R, Rachlis M (2008). Strategies for physicians to mitigate the health effects of poverty. Semantic Scholar.

[16] Weintraub D, Rodgers MA, Botcheva L, Loeb A, Knight R, Ortega K, Heymach B, Sandel M, Huffman L (2010). Pilot study of medical-legal partnership to address social and legal needs of patients. J Health Care Poor Underserved.

[17] Cohen E, Fullerton DF, Retkin R, Weintraub D, Tames P, Brandfield J, Sandel M (2010). Medical-legal partnership: collaborating with lawyers to identify and address health disparities. J Gen Intern Med.

[18] Lawton EM, Sandel M (2014). Investing in legal prevention: connecting access to civil justice and healthcare through medical-legal partnership. J Leg Med.

[19] Whitehead M, Dahlgren G (2006). Levelling up (part 1)‎ : a discussion paper on concepts and principles for tackling social inequities in health / by Margaret Whitehead and Göran Dahlgren. World Health Organization.

[20] Marchis EH, Brown E, Aceves BA, Loomba V, Molina M, Cartier Y, Wing H, Gottlieb LM (2022). SCREEN report: state of the science on social screening in healthcare settings. Social Interventions Research & Evaluation Network.

[21] Sharma M, Pinto AD, Kumagai AK (2018). Teaching the social determinants of health: a path to equity or a road to nowhere?. Acad Med.

[22] Komeiha M, Kujbida G, Reynolds A, Mbagwu I, Dojeiji L, O'Rourke JJ, Raju S, Varia M, Stylianou H, Burgess S, Ogundele OJ, Pinto AD (2024). A study of the enablers and barriers to the collection of sociodemographic data by public health units in Ontario, Canada during the COVID-19 pandemic. BMC Public Health.

[23] Kiran T, Pinto AD (2016). Swimming "upstream" to tackle the social determinants of health. BMJ Qual Saf.

[24] Gottlieb L, Sandel M, Adler NE (2013). Collecting and applying data on social determinants of health in health care settings. JAMA Intern Med.

[25] Gottlieb L, Tobey R, Cantor J, Hessler D, Adler NE (2016). Integrating social and medical data to improve population health: opportunities and barriers. Health Aff (Millwood).

[26] Wallace AS, Luther BL, Sisler SM, Wong B, Guo J (2021). Integrating social determinants of health screening and referral during routine emergency department care: evaluation of reach and implementation challenges. Implement Sci Commun.

[27] Aery A, Rucchetto A, Singer A, Halas G, Bloch G, Goel R, Raza D, Upshur RE, Bellaire J, Katz A, Pinto AD (2017). Implementation and impact of an online tool used in primary care to improve access to financial benefits for patients: a study protocol. BMJ Open.

[28] Cook LA, Sachs J, Weiskopf NG (2021). The quality of social determinants data in the electronic health record: a systematic review. J Am Med Inform Assoc.

[29] Morse DF, Sandhu S, Mulligan K, Tierney S, Polley M, Chiva Giurca B, Slade S, Dias S, Mahtani KR, Wells L, Wang H, Zhao BO, De Figueiredo CE, Meijs JJ, Nam HK, Lee KH, Wallace C, Elliott M, Mendive JM, Robinson D, Palo M, Herrmann W, Østergaard Nielsen R, Husk K (2022). Global developments in social prescribing. BMJ Glob Health.

[30] Davis VH, Qiang JR, Adekoya MacCarthy I, Howse D, Seshie AZ, Kosowan L, Delahunty-Pike A, Abaga E, Cooney J, Robinson M, Senior D, Zsager A, Aubrey-Bassler K, Irwin M, Jackson LA, Katz A, Marshall EG, Muhajarine N, Neudorf C, Garies S, Pinto AD (2025). Perspectives on using artificial intelligence to derive social determinants of health data from medical records in Canada: large multijurisdictional qualitative study. J Med Internet Res.

[31] Davis VH, Rodger L, Pinto AD (2023). Collection and use of social determinants of health data in inpatient general internal medicine wards: a scoping review. J Gen Intern Med.

[32] Davis VH, Dainty KN, Dhalla IA, Sheehan KA, Wong BM, Pinto AD (2023). "Addressing the bigger picture": a qualitative study of internal medicine patients' perspectives on social needs data collection and use. PLoS One.

[33] Henrikson NB, Blasi PR, Dorsey CN, Mettert KD, Nguyen MB, Walsh-Bailey C, Macuiba J, Gottlieb LM, Lewis CC (2019). Psychometric and pragmatic properties of social risk screening tools: a systematic review. Am J Prev Med.

[34] Karran EL, G Cashin AG, Barker T, A Boyd M, Chiarotto A, Dewidar O, Petkovic J, Sharma S, Tugwell P, Moseley GL, Identifying Social Factors that Stratify Health Opportunities and Outcomes (ISSHOOs) Collaborative Core Research Group (2023). The ' and '' of screening for social needs in healthcare settings: a scoping review. PeerJ.

[35] Broaddus-Shea ET, Fife Duarte K, Jantz K, Reno J, Connelly L, Nederveld A (2022). Implementing health-related social needs screening in western Colorado primary care practices: qualitative research to inform improved communication with patients. Health Soc Care Community.

[36] Drake C, Batchelder H, Lian T, Cannady M, Weinberger M, Eisenson H, Esmaili E, Lewinski A, Zullig LL, Haley A, Edelman D, Shea CM (2021). Implementation of social needs screening in primary care: a qualitative study using the health equity implementation framework. BMC Health Serv Res.

[37] Adekoya I, Delahunty-Pike A, Howse D, Kosowan L, Seshie Z, Abaga E, Cooney J, Robinson M, Senior D, Zsager A, Aubrey-Bassler K, Irwin M, Jackson L, Katz A, Marshall E, Muhajarine N, Neudorf C, Pinto AD (2023). Screening for poverty and related social determinants to improve knowledge of and links to resources (SPARK): development and cognitive testing of a tool for primary care. BMC Prim Care.

[38] Pinto AD, Glattstein-Young G, Mohamed A, Bloch G, Leung F, Glazier RH (2016). Building a foundation to reduce health inequities: routine collection of sociodemographic data in primary care. J Am Board Fam Med.

[39] Pinto AD, Shenfeld E, Lattanzio R, Aratangy T, Wang R, Nisenbaum R, Kiran T (2020). Routine identification of patients with disabilities in primary care: a mixed-methods study. Disabil Health J.

[40] Kiran T, Sandhu P, Aratangy T, Devotta K, Lofters A, Pinto AD (2019). Patient perspectives on routinely being asked about their race and ethnicity: qualitative study in primary care. Can Fam Physician.

[41] Pinto AD, Aratangy T, Abramovich A, Devotta K, Nisenbaum R, Wang R, Kiran T (2019). Routine collection of sexual orientation and gender identity data: a mixed-methods study. CMAJ.

[42] The Scottish Deep End Project - about us. University of Glasgow.

[43] Watt G, Brown G, Budd J, Cawston P, Craig M, Jamieson R, Langridge S, Lyon A, Mercer S, Morton C, Mullin A, O'Neil J, Paterson E, Sambale P, Watt G, Williamson A (2012). General practitioners at the deep end: the experience and views of general practitioners working in the most severely deprived areas of Scotland. Occas Pap R Coll Gen Pract.

[44] Sturgiss E, Tait PW, Douglas K, Chew J, Baglow S, Watt G (2019). GPs at the deep end: identifying and addressing social disadvantage wherever it lies. Aust J Gen Pract.

[45] Walton L, Ratcliffe T, Jackson BE, Patterson D (2016). Mining for deep end GPs: a group forged with steel in Yorkshire and Humber. Br J Gen Pract.

[46] Steen R, Walton E, Patterson D (2020). Jumping in at the deep end: supporting young GPs working in deprivation. Br J Gen Pract.

[47] Watt G General practitioners at the deep end international bulletin No 11. University of Glasgow.

[48] Primary health care at the deep end Canada. Deep End Canada.

[49] (2013). We ask because we care: the tri-hospital + TPH health equity data collection research project report. Toronto Public Health.

[50] Williams-Roberts H (2017). We ask because we care: feasibility and acceptability of sociodemographic data collection in Saskatoon. University of Saskatchewan.

[51] Proctor E, Silmere H, Raghavan R, Hovmand P, Aarons G, Bunger A, Griffey R, Hensley M (2011). Outcomes for implementation research: conceptual distinctions, measurement challenges, and research agenda. Adm Policy Ment Health.

[52] Damschroder LJ, Aron DC, Keith RE, Kirsh SR, Alexander JA, Lowery JC (2009). Fostering implementation of health services research findings into practice: a consolidated framework for advancing implementation science. Implement Sci.

[53] Damschroder LJ, Reardon CM, Widerquist MA, Lowery J (2022). The updated consolidated framework for implementation research based on user feedback. Implement Sci.

[54] Glasgow RE, Vogt TM, Boles SM (1999). Evaluating the public health impact of health promotion interventions: the RE-AIM framework. Am J Public Health.

[55] McCreight MS, Rabin BA, Glasgow RE, Ayele RA, Leonard CA, Gilmartin HM, Frank JW, Hess PL, Burke RE, Battaglia CT (2019). Using the Practical, Robust Implementation and Sustainability Model (PRISM) to qualitatively assess multilevel contextual factors to help plan, implement, evaluate, and disseminate health services programs. Transl Behav Med.

[56] Miles MB, Huberman AM (1994). Qualitative Data Analysis: An Expanded Sourcebook.

[57] Resources - Deep End Canada. Deep End Canada.

[58] Hunik L, Sturgiss E, Terry A, Blane D, Eggleton K, Maharaj R, Tane T, Olde Hartman T, Drinkwater J, Gabet M, Hauck FR, Henry M, Mamo N, Wallace R, Klein D (2024). The role of the primary healthcare research community in addressing the social and structural determinants of health: a call to action from NAPCRG 2023. Fam Med Community Health.

[59] Skivington K, Matthews L, Simpson SA, Craig P, Baird J, Blazeby JM, Boyd KA, Craig N, French DP, McIntosh E, Petticrew M, Rycroft-Malone J, White M, Moore L (2021). A new framework for developing and evaluating complex interventions: update of Medical Research Council guidance. BMJ.

[60] Primary care needs OurCare: the final report of the largest pan-Canadian conversation about primary care. MAP Centre for Urban Health Solutions.

[61] Kiran T, Thelen R, Szymanski K, Daneshvarfard M, Rajendra KL, Lim J, Garabet M, Mayer L, Black S, Waterbury JD, Katz A, Condon A, Lavergne MR, Stringer K, Breton M, Kovacina N, Buss M, Kay J, MacLeod P, Mitra G (2025). Public priorities for primary care in Canada: report on insights and actionable recommendations from 5 provincial reference panels. Can Fam Physician.

[62] OurCare needs everyone — including you. OurCare.

[63] (2025). Bill 13, An Act respecting primary care, Royal Assent, 44th Leg, Ontario.

