# Supplementary material for: Advancing Health Equity Through Primary Care: Protocol for the Spread, Scale, and Multimethod Developmental Evaluation of the Deep End Canada Network
Source: JMIR Res Protoc. 2025 Oct 10;14:e75732. doi: 10.2196/75732 (PMC12552831; doi:10.2196/75732)
Supplement: Multimedia Appendix 2 [file resprot_v14i1e75732_app2.pdf]

SPARK Reach and Adoption Survey

This survey will help us better understand the reach and adoption of the SPARK Tool across Canada. The SPARK Tool is a 20-item questionnaire about patient's demographics and social needs for use in primary care, and serves as a gold standard across Canada[1]. The SPARK Tool includes questions about demographic information (e.g., preferred language, immigration status, Indigenous identity, race, disability status, sex at birth and gender identity, sexual orientation) and social needs (education, income, medication access, housing status, social isolation, transportation, cost of utilities, and precarious employment).

This survey consists of three parts and should take approximately 10-20 minutes to complete:

1. Participant information
2. SPARK Reach and Adoption Survey
3. Optional contact details for follow-up

After each part, you will be automatically redirected to the next part.

Please coordinate within your team to gather responses from the appropriate staff and **submit one survey response per clinic**. Findings will inform the development of recommendations to support demographic and social needs collection and use across Canada.

This study was approved by the Unity Health Toronto Research Ethics Board [REB #24-094]. Information will be collated by staff at Upstream Lab. Responses will remain securely stored and only accessed by those involved in this research project.

Before completing this survey, please review the Information Letter and Consent for Survey document [link]. If you have any questions about the consent information or survey, please contact Upstream Lab (upstreamlab@unityhealth.to).

Consent Information

The research study has been explained to me, and any questions I had have been answered to my satisfaction. I have the right not to participate and the right to withdraw without affecting my professional, academic standing or care provided to me at St. Michael's Hospital. As well, the potential risks and benefits of participating in this research study have been explained to me.

I have not waived my legal rights nor released the investigators, sponsors, or involved institutions from their legal and professional responsibilities. I know that I may ask now, or in the future, any questions I have about the study. I have been told that records relating to me and my care will be kept confidential and that no information will be disclosed without my

[1] Adekoya, I., Delahunty-Pike, A., Howse, D. et al. Screening for poverty and related social determinants to improve knowledge of and links to resources (SPARK): development and cognitive testing of a tool for primary care. *BMC Prim. Care* **24**, 247 (2023). https://doi.org/10.1186/s12875-023-02173-8

permission unless required by law. I have been given sufficient time to read the above information.

By completing and submitting this survey, your free and informed consent is implied and indicates that you understand the above statement and conditions of participation in this study.

Section A: Background

1. Please provide your name and email address:
    a. Name:
    b. Email address:
2. In which province or territory do you currently reside and work?
    a. AB
    b. BC
    c. SK
    d. MB
    e. ON
    f. QC
    g. NB
    h. NL
    i. NS
    j. PE
    k. YT
    l. NU
    m. NT
3. What is the name of the individual clinic and/or larger organization (e.g., network of clinics) that you represent?
    a. Clinic name (e.g., 61 Queen Clinic):
    b. Larger network/organization name (e.g., St. Michael's Academic Family Health Team):
4. Are you completing this survey on behalf of the individual clinic or the larger network/organization?
    a. On behalf of the individual clinic
    b. On behalf of the larger network/organization
5. What type of organization do you represent? Please check all that apply.
    a. Primary Care
    b. Hospital
    c. Long-term care
    d. Mental health care
    e. Public health
    f. Pharmacy
    g. Research
    h. Social services or community organization

   i. Health policy
   j. Funding agency or organization
   k. Other type of organization. Please specify:

6. What role(s) do you hold at your clinic and/or organization? Please check all that apply.
   a. Health care provider
   b. Social services provider
   c. Researcher/evaluator
   d. Administrator
   e. Policymaker
   f. Other role. Please specify:

Section B: Organization / Clinic Background

7. Do you work at a clinic and/or organization providing healthcare services to patients? Y/N
   a. If yes: Is your clinic and/or organization part of a network of other clinics/organizations? Y/N Please enter a number or "unknown":
      i. How many other clinics are in your organization?
      ii. How many other clinics in your organization intend to administer the SPARK Tool?
      iii. How many other clinics in your organization currently administer the SPARK Tool?
   b. If yes: What is the total number of patients (e.g., registered or rostered) at your clinic and/or organization?
   c. If yes: Of that total number, how many patients are active (i.e., number of patients with a visit in the last 2 years) at your clinic and/or organization?
8. Does your clinic and/or organization have an explicit focus to serve patients with social needs (e.g., precarious housing, low income, food insecurity, equity deserving populations, patients made vulnerable by social and economic policies, facing systemic barriers to care, and/or experiencing poverty)? Y/N

Section C: Familiarity and Experience with Demographic and Social Needs Questions

9. Do you currently or did you previously <u>collect</u> demographic and social needs data at your clinic and/or organization?
      i. Yes, we <u>currently</u> collect demographic and social needs data.
      ii. Yes, we <u>previously</u> collected demographic and social needs data.
      iii. No, we <u>do not currently collect and never previously collected</u> demographic and social needs data.
   a. If yes, currently:

a. What demographic and social needs data does your clinic and/or organization <u>currently</u> collect? Please select all that apply (and, if needed, add any additional context into the “Other. Please describe:” text box):
    i. Demographic data (standardized; e.g., survey). Please input name of survey(s)/tool(s):
    ii. Demographic data (non-standardized; e.g., social screening interview).
    iii. Social needs data (standardized). Please input name of survey(s)/tool(s):
    iv. Social needs data (non-standardized).
    v. Other. Please describe:
b. How does your clinic and/or organization <u>currently</u> collect the demographic and social needs data? Please select all that apply (and, if needed, add any additional context into the “Other. Please describe:” text box):
    i. Electronic survey via email or secure messaging (patients self-report).
    ii. Electronic survey via QR Code on clinic poster (patients self-report).
    iii. Paper format survey (patients self-report).
    iv. Clerical staff collect.
    v. Some providers collect (e.g., social workers, nurses).
    vi. Specific providers collect (e.g., Community Health Worker)
    vii. All providers collect.
    viii. Other. Please describe:
c. How frequently does your clinic and/or organization <u>currently</u> collect the demographic and social needs data? Please select all that apply (and, if needed, add any additional context into the “Other. Please describe:” text box):
    i. Once at intake.
    ii. Multiple times per year.
    iii. Once per year.
    iv. Once every two or more years.
    v. As needed. Please describe:
    vi. Other. Please describe:
d. Does your clinic and/or organization <u>currently use</u> the collected demographic and social needs data? Y/N
    i. If yes: How does your clinic and/or organization <u>currently use</u> the demographic and social needs data? Please select all that apply (and, if needed, add any additional context into the “Other. Please describe:” text box):
        1. At intake to create patient charts.
        2. Improve individual care.
        3. Make patient referrals (e.g., to a social resource)
        4. Quality improvement.
        5. Program planning.
        6. Institutional reporting purposes

7. Particular appointment types (e.g., social needs).
8. Other. Please describe:

b. If yes, previously:

   a. What demographic and social needs data has your clinic and/or organization <u>previously</u> collected? Please select all that apply (and, if needed, add any additional context into the “Other. Please describe:” text box):
      i. Demographic data (standardized; e.g., questionnaire). Please input name of survey(s)/tool(s):
      ii. Demographic data (non-standardized; e.g., social screening interview).
      iii. Social needs data (standardized). Please input name of survey(s)/tool(s):
      iv. Social needs data (non-standardized).
      v. Other. Please describe:

   b. How did your clinic and/or organization <u>previously</u> collect the demographic and social needs data? Please select all that apply (and, if needed, add any additional context into the “Other. Please describe:” text box):
      i. Electronic survey via email or secure messaging (patients self-report).
      ii. Electronic survey via QR Code on clinic poster (patients self-report).
      iii. Paper format survey (patients self-report).
      iv. Clerical staff collected.
      v. Some providers collected (e.g., social workers, nurses).
      vi. Specific providers collected (e.g., Community Health Worker)
      vii. All providers collected.
      viii. Other. Please describe:

   c. How frequently did your clinic and/or organization <u>previously</u> collect the demographic and social needs data? Please select all that apply (and, if needed, add any additional context into the “Other. Please describe:” text box):
      i. Once at intake.
      ii. Multiple times per year.
      iii. Once per year.
      iv. Once every two or more years.
      v. As needed. Please describe:
      vi. Other. Please describe:

   d. Did your clinic and/or organization <u>previously use</u> the collected demographic and social needs data? Y/N
      i. If yes: How did your clinic and/or organization <u>previously use</u> the demographic and social needs data? Please select all that apply (and, if needed, add any additional context into the "Other. Please describe:" text box):
         1. At intake to create patient charts.
         2. Improve individual care.
         3. Make patient referrals (e.g., to a social resource)

4. Quality improvement.
5. Program planning.
6. Institutional reporting purposes.
7. Particular appointment types (e.g., social needs).
8. Other. Please describe:

10. Demographic and social needs questions, like those asked in the SPARK Tool, are also used in similar surveys such as the Health Equity Questionnaire or Measuring Health Equity Survey. Have you used any such surveys similar to the SPARK Tool? Y/N
    a. If yes: What was the survey called when you received it?
    b. If yes: From where did you receive or hear about the survey?

Section D: Actual or Intended Adoption of the SPARK Tool

11. Have you administered the SPARK Tool to patients at your clinic and/or organization? Y/N
    b. If yes: Do you currently administer the SPARK Tool to patients at your clinic and/or organization? Y/N
        i. If yes: Please select how you currently administer the SPARK Tool to patients. Please select all that apply (and, if needed, add any additional context into the "Other. Please describe:" text box):
            1. Electronic survey via email or secure messaging (self-administer).
            2. Electronic survey via QR Code on clinic poster (self-administer).
            3. Paper format (self-administer).
            4. Clerical staff administer survey.
            5. Some providers administer survey (e.g., social workers, nurses).
            6. Specific providers administer survey (e.g., Community Health Worker)
            7. All providers administer survey.
            8. Other. Please describe:
        ii. If yes: Please select how you currently promote or provide information about the SPARK Tool at your clinic and/or organization. Please select all that apply (and, if needed, add any additional context into the "Other. Please describe:" text box):
            1. Emails to patients
            2. Clinic posters
            3. Newsletters
            4. Clerical staff asked to offer survey to patients
            5. Providers asked to offer survey to patients
            6. Other. Please describe:
        iii. If yes: Please select how you currently use data from SPARK Tool responses at your clinic and/or organization. Please select all that apply

(and, if needed, add any additional context into the “Other. Please describe:” text box):
1. At intake to create patient charts.
2. Improve individual care.
3. Make patient referrals (e.g., to a social resource)
4. Quality improvement.
5. Program planning.
6. Institutional reporting purposes
7. Particular appointment types (e.g., social needs).
8. Other. Please describe:

c. If no: Do you intend to administer the SPARK Tool to patients at your clinic and/or organization? Y/N
   i. If yes: Please select how you currently intend to administer the SPARK Tool to patients. Please select all that apply (and, if needed, add any additional context into the “Other. Please describe:” text box):
      1. Electronic survey via email or secure messaging (self-administer).
      2. Electronic survey via QR Code on clinic poster (self-administer).
      3. Paper format (self-administer).
      4. Clerical staff to administer survey.
      5. Some providers to administer survey (e.g., social workers, nurses).
      6. Specific providers to administer survey (e.g., Community Health Worker)
      7. All providers to administer survey.
      8. Other. Please describe:
   ii. If yes: Please select how you currently intend to promote or provide information about the SPARK Tool at your clinic and/or organization. Please select all that apply (and, if needed, add any additional context into the “Other. Please describe:” text box):
      1. Emails to patients
      2. Clinic posters
      3. Newsletters
      4. Clerical staff to offer survey to patients
      5. Providers to offer survey to patients
      6. Other. Please describe:
   iii. If yes: Please select how you currently intend to use data from SPARK Tool responses at your clinic and/or organization. Please select all that apply (and, if needed, add any additional context into the “Other. Please describe:” text box):
      1. At intake to create patient charts.
      2. Improve individual care.
      3. Make patient referrals (e.g., to a social resource)
      4. Quality improvement.
      5. Program planning.

6. Institutional reporting purposes
7. Particular appointment types (e.g., social needs).
8. Other. Please describe:

12. Do you have any other comments or feedback that you would like to share with the SPARK Study team regarding the above survey or your experiences and thoughts on the collection and use of demographic and social needs data in your clinic and/or organization?

Section E: Consent to Contact for Follow-up Survey and Research

13. We would like to follow-up with another 10-20 minute survey in approximately 6 months' time, as part of our SPARK study evaluating the reach, adoption, and implementation of the SPARK Tool across Canada. Do you consent to receive an invitation to participate in a follow-up survey? Y/N
    a. If yes: Should we contact the email address provided at the beginning of this survey for the follow-up survey? Y/N
        i. If no: Please provide a more appropriate name and email address for us to contact:

Thank you for taking the time to complete this survey. You will be able to access the SPARK Tool resources after you click next.

Draft SPARK Reach and Adoption Survey 2 (Follow-up Questions)

Thank you for completing the first SPARK Reach and Adoption Survey. In this Follow-up Survey, we are interested in learning more about changes in the reach and adoption of the SPARK Tool across Canada since the first survey.

If you no longer work at the clinic/organization on behalf of which you completed the first Reach and Adoption Survey, please send this survey link to the most appropriate person.

The SPARK Tool is a 17 (plus two optional) item questionnaire about patient's demographics and social needs for use in primary care, and serves as a standard across Canada. The SPARK Tool includes questions about demographic information (e.g., preferred language, immigration status, Indigenous identity, race, disability status, gender identity, and sexual orientation; optional questions include ethnicity and religion) and social needs (e.g., education, income, medication access, housing status, social isolation, transportation, cost of utilities, and precarious employment).

This survey consists of four parts and should take approximately 10-20 minutes to complete. The survey will ask you follow up questions regarding the implementation of SPARK Tool in your clinic or organizations:

1. Clinic and/or organization background
2. Deep End Canada resources
3. Familiarity and experience with demographic and social needs questions
4. SPARK Tool

Please coordinate within your team to gather responses from the appropriate staff and submit one survey response per clinic.

Findings will inform the development of recommendations to support demographic and social needs collection and use across Canada. This study was approved by the Unity Health Toronto Research Ethics Board [REB #24-094]. Information will be collated by staff at Upstream Lab. Responses will remain securely stored and only accessed by those involved in this research project.

Before completing this survey, please review the Information Letter and Consent for Survey. If you have any questions about the consent information or survey, please contact Upstream Lab (upstreamlab@unityhealth.to).

Consent Information

The research study has been explained to me, and any questions I had have been answered to my satisfaction. I have the right not to participate and the right to withdraw without affecting my professional, academic standing or care provided to me at St. Michael's Hospital. As well, the potential risksand benefits of participating in this research study have been explained to me.

I have not waived my legal rights nor released the investigators, sponsors, or involved institutions from their legal and professional responsibilities. I know that I may ask now, or in

the future, any questions I have about the study. I have been told that records relating to me and my care will be kept confidential and that no information will be disclosed without my permission unless required by law. I have been given sufficient time to read the above information.

By completing and submitting this survey, your free and informed consent is implied and indicates that you understand the above statement and conditions of participation in this study.

Section A: Clinic and Organization Background

1. **Do you still work at the clinic /organization where you were working when you completed the first Reach and Adoption survey?**
    - **If not, who should we contact at that clinic/organization?**
2. **Are you completing this survey on behalf of an individual clinic or a larger network/organization?**
    - On behalf of individual clinic (e.g., 61 Queen St E Clinic)
        i. **Please provide the name of the clinic:**
    - On behalf of the larger network/organization (e.g., St Michael's Hospital Academic Family Health Team)
        i. **Please provide the name of the larger network/organization:**
        ii.
3. **Has your role, job title, or involvement in providing healthcare services changed since you last completed the survey?**
    - Yes
    - No
        i. If yes, choose one: health care provider, social services provider, researchers/ evaluator, administrator, policy-maker, other role (text box)

Section B: Deep End Canada Resources

4. **Did you access the materials and resources for demographic and social needs data collection and use created by Primary Health Care at the Deep End Canada [link]?**
    - Yes
    - No
5. **If yes, which resources did you use?**
    - Please describe:
6. **If yes, please describe briefly how you used these resources?**

Section C: Familiarity and Experience with Demographic and Social Needs Questions

7. **Do you currently *collect* demographic and social needs data at your clinic and/or organization?**
    - Yes, we currently collect demographic and social needs data
    - No, but we previously collected demographic and social needs data and stopped
    - No, we do not currently collect and never previously collected demographic and social needs data
8. **If no, could you elaborate on the reasons for stopping and/or barriers to collecting demographic and social needs data at your clinic and/or organization?**
9. **Has your clinic and/or organization made any changes in how it collects or uses demographic and social needs data since you last completed the survey?**
    - Yes, we have started collecting using the SPARK Tool only
    - Yes, we have expanded or improved our data collection by using the SPARK Tool and/or other toolsNo, our approach has remained the same
10. **If yes, what demographic and social needs data does your clinic and/or organization currently *collect*? Please select all that apply (and, if needed, add any additional context into the "Other. Please describe:" text box):**
    - Demographic data (standardized; e.g., survey). Please input name of survey(s)/tool(s): (text box)
    - Demographic data (non-standardized; e.g., new patient intake survey)
    - Social needs data (standardized). Please input name of survey (s)/tool (s): (text box)
    - Social needs data (non-standardized)
    - Other. Please describe: (text box)
11. **If yes, how does your clinic and/or organization currently *collect* the demographic and social needs data? Please select all that apply (and, if needed, add any additional context into the "Other. Please describe:" text box):**
    - Electronic survey via email or secure messaging (patients self-report)
    - Electronic survey via QR Code on clinic poster (patients self-report)
    - Paper format survey (patients self report)
    - Clerical staff collect
    - Some providers collect

- Only specific providers collect
- All providers collect
- Other. Please describe: (text box)

12. **If yes, how frequently does your clinic and/or organization currently *collect* the demographic and social needs data? Please select all that apply (and, if needed, add any additional context into the "Other. Please describe:" text box):**
    - Once at intake
    - Multiple times per year
    - Once per year
    - Once every two or more years
    - As needed. Please describe: (text box)
    - Other. Please describe: (text box)
13. **Does your clinic and/or organization currently *use* the collected demographic and social needs data?**
    - Yes
    - No
14. **If yes, how does your clinic and/or organization currently use the demographic and social needs data? Please select all that apply (and, if needed, add any additional context into the "Other. Please describe:" text box):**
    - At intake to create patient charts
    - Improve individual care
    - Make patient referrals (e.g., to a social resource)
    - Quality improvement
    - Program planning
    - Institutional reporting purposes
    - Particular appointment types (e.g., social needs)
    - Other. Please describe: (text box)
15. **Demographic and social needs questions, like those asked in the SPARK Tool, are also used in similar surveys such as the Health Equity Questionnaire or Measuring Health Equity Survey. Since you last completed our survey, have you started using any new surveys similar to the SPARK Tool?**
    - Yes (please state the name of each survey text box)
    - No, but we started using the SPARK Tool
    - No, but we continued using the SPARK Tool
    - No, we are using the same surveys as before (which may include the SPARK Tool)
    - Other (text box)

Section D: SPARK Tool

16. **Have you administered the SPARK Tool to patients at your clinic and/or organization (validated 2024 version (link))?**
    - Yes

- No

17. If yes, did you make any modifications or only use some questions?
- Yes
- No

18. If yes, please describe any changes made or specify which questions you used?
- (text box)

19. How was your experience using the SPARK tool? In your answer, please describe any resources or support that you have used and how they have helped or not.
- (text box)

20. If yes, please select how you currently administer the SPARK Tool to patients. Please select all that apply (and, if needed, add any additional context into the "Other. Please describe:" text box):
- Electronic survey via email or secure messaging (patients self-report)
- Electronic survey via QR Code on clinic poster (patients self-report)
- Paper format (patients self-report)
- Clerical staff administer survey
- Some providers administer survey
- Only specific providers administer survey
- All providers administer survey
- Other. Please describe: (text box)

21. If yes, please select how you currently promote or provide information about the SPARK Tool at your clinic and/or organization. Please select all that apply (and, if needed, add any additional context into the "Other. Please describe:" text box):
- Emails to patients
- Clinic posters
- Newsletters
- Clerical staff are asked to offer survey to patients
- Providers are asked to offer survey to patients
- We do not promote or provide information about the SPARK Tool or patients learn about the SPARK Tool when they complete it
- Other. Please describe:

22. If yes, please select how you currently use data from SPARK Tool responses at your clinic and/or organization. Please select all that apply (and, if needed, add any additional context into the "Other. Please describe:" text box):
- At intake to create patient charts
- Improve individual care
- Make patient referrals (e.g., to a social resource)
- Quality improvement
- Program planning
- Institutional reporting purposes
- Particular appointment types (e.g., social needs

- Other. Please describe: (text box)

23. **Have you used the French version [link] of the SPARK Tool?**
    - Yes
    - No
24. **Does your clinic or organization need any additional resources or information to support your use of the SPARK Tool? Please select all that apply. (If yes, we will contact you to schedule a time for a brief meeting).**
    - Yes, we would like additional resources
    - Yes, we would like more information
    - No
25. **Primary Health Care at the Deep End Canada is a pan-Canadian network of primary health care teams, including health professionals, researchers, patient partners, and decision-makers with the same goal: address social determinants of health and advance health equity at the individual, clinic, and policy levels. Members receive resources and personalized coaching support. Are you interested in learning more about membership to Deep End Canada?**
    - Yes
    - No
26. **Do you have any other comments or feedback that you would like to share with the SPARK Study team regarding the above survey or your experiences and thoughts on the collection and use of demographic and social needs data in your clinic and/or organization?**
    - (text box)
