# Supplementary material for: Advancing Health Equity Through Primary Care: Protocol for the Spread, Scale, and Multimethod Developmental Evaluation of the Deep End Canada Network
Source: JMIR Res Protoc. 2025 Oct 10;14:e75732. doi: 10.2196/75732 (PMC12552831; doi:10.2196/75732)
Supplement: Multimedia Appendix 3 [file resprot_v14i1e75732_app3.pdf]

Draft Focus Group and Interview Guide for Deep End Canada – Members

Preamble: My name is [Research Staff Name] and I'm a [Role] with the Upstream Lab at St Michael's Hospital. Primary Health Care at the Deep End Canada is a network of primary health care teams, including health professionals, researchers, patient partners, and decision-makers, working with patients who may face social and/or economic disadvantages. Our mission is to address health equity by improving the collection and use of social data to address social determinants of health in primary health care at the individual, clinic, and policy levels and share ideas and projects across Canadian primary health care.

Deep End Canada is guided by three pillars: Data-Driven Local Solutions, Social Action and Advocacy, and Community and Key Actor Engagement, and we are using these to guide our questions today. We are a new network that started in June 2024 and are interested in your perspectives regarding your involvement as well as your recommendations for the future.

Today's focus group will be structured more like a conversation, using our questions as a guide. We are not evaluating you or your clinic in any way, and you do not need to share any private information about you, your patients or your clinic. However, none of what is shared will be attributed to you as an individual (the transcript will be de-identified) and we encourage you to be honest in your feedback, critiques, or questions.

Participation in Deep End Canada

1. How did you get involved in Deep End Canada?
    a. *Prompt*: Where did you learn about it?

2. We've been working with you since [insert date] as part of Deep End Canada. Can you provide a broad overview of your activities during that time?
    a. What inspired you to participate in Deep End Canada?

Pillars 1 & 3: Data-Driven Local Solutions; Social Action and Advocacy

3. Can you describe how your clinic currently uses the SPARK tool and/or other demographic and social needs data tools to collect and use social data in your patient care and decision-making?
    a. *Prompt*: Can you walk us through the process or workflow of collecting, analyzing and using social data for decision-making at your clinic?

4. If you have done any analysis on the data, what are some examples of common social needs or any patterns you have noticed regarding the individuals/communities your organization serves?

5. Can you describe how your internal clinic organizational structures, policies, and resources **support** social data collection and use?
    a. Can you describe how they **hinder** social data collection and use?

6. Can you describe how external structures, policies, and resources **support** social data collection and use?
    a. Can you describe how they **hinder** social data collection and use?
    b. *Prompt*: E.g., Financial resources to implement or update EMR systems for data collection, policy changes within your health system or province.

7. One of the Deep End Canada core pillars is Social Action and Advocacy. A priority for primary health care is a focus beyond individual patients to the health of communities, which can require upstream changes. How does your organization define and engage in advocacy?
    a. What are examples, if any, of advocacy activities in which you or your organization engage?
    b. Based on your definition of advocacy, what role should Deep End Canada play in advocacy towards advancing health equity and addressing social determinants of health?

8. Deep End Canada offers support to primary health care teams through facilitating pan-Canadian network meetings, individual coaching meetings, and offering materials and resources.
    a. To what extent have you used one or more of these three supports?
        i. *Prompt*: If you used the materials and resources, how did these help you?
        ii. *Prompt*: If you used the Change Package [a PDF document providing recommendations from the SPARK Tool Implementation study], how did it help you?
    b. Can you provide examples of how they have helped you?
    c. What other supports would be useful?

9. What other supports have you used outside of Deep End Canada to support your work in [insert activities that participants previously discussed; e.g., data collection, use, and advocacy]?
    a. Can you describe the impact of these supports in aiding your organization's collection and use of social data to better care for patients?
    b. What other impacts have these supports had on your overall work caring for patients and addressing social needs within your community?

Pillar 2: Community and Key Actor Engagement

Now – we are interested in your experience being part of Deep End Canada and your work on community engagement. We define community engagement as "*involving communities in decision-making and in the planning, design, governance and delivery of services for said communities*", but let us know if your understanding or definition is different from ours.

10. What does community engagement look like at your organization?
    a. *Prompt:* What strategies have you used in your work?
    b. *Prompt:* Which communities do you engage through these strategies (e.g., patients, community organizations)?
        i. [Prompt for: ideas/definitions of what community organizations are; descriptions of the populations that are engaged]
    c. *Prompt:* Who plays the most significant role in community engagement at your organization?
        i. [Prompt for: the role of senior leadership, patient and community partners, which departments or projects do more or less community engagement, etc.]

11. What community engagement have you done around the collection and use of demographic and social needs data?

12. The IAP2 Framework is one framework for identifying the level of public involvement in decision making processes. When thinking about your community engagement activities, where would you rate them on the IAP2 framework? Please share why you ranked your engagement that way.

INCREASING IMPACT ON THE DECISION

	INFORM	CONSULT	INVOLVE	COLLABORATE	EMPOWER
PUBLIC PARTICIPATION GOAL	To provide the public with balanced and objective information to assist them in understanding the problem, alternatives, opportunities and/or solutions.	To obtain public feedback on analysis, alternatives and/or decisions.	To work directly with the public throughout the process to ensure that public concerns and aspirations are consistently understood and considered.	To partner with the public in each aspect of the decision including the development of alternatives and the identification of the preferred solution.	To place final decision making in the hands of the public.
PROMISE TO THE PUBLIC	We will keep you informed.	We will keep you informed, listen to and acknowledge concerns and aspirations, and provide feedback on how public input influenced the decision.	We will work with you to ensure that your concerns and aspirations are directly reflected in the alternatives developed and provide feedback on how public input influenced the decision.	We will look to you for advice and innovation in formulating solutions and incorporate your advice and recommendations into the decisions to the maximum extent possible.	We will implement what you decide.

© IAP2 International Federation 2018. All rights reserved. 20181112_v1

13. In what ways do you think your current community engagement strategies could improve?

14. How has Deep End Canada supported your work related to community engagement?
    a. *Prompt*: Can you provide specific examples?

Sustainability and Future of Deep End Canada

Deep End Canada is currently being facilitated by staff at the Upstream Lab, as we have received grant funding for its operations until March, 2027. However, we are hoping that the network continues and grows beyond this research project.

15. Our three core pillars include Data-driven local solutions, community and key actor engagement, and social action and advocacy. We intend to be a member-driven organization that reflects the material priorities of those working in and receiving primary health care. Are these pillars reflective of your experience working within Deep End Canada?

16. Thinking about Deep End Canada's future, what would you change about these pillars, if anything?
    a. *Prompt*: Show corresponding activities under pillars if needed.

17. What do you think is the impact of us being a pan-Canadian network compared to more local groups?
    a. *Prompt*: How does participation in Deep End Canada complement or differ from support received from other networks, such as local/regional Community of Practice groups?

18. What factors would ensure the sustainability and continuation of Deep End Canada?
    a. *Prompt*: What kind of knowledge or skillset should future facilitators have?

19. Which pan-Canadian organization (e.g., College of Family Physicians) would be appropriate to take over the facilitation of the Deep End Canada network after our funding ends?

Wrap Up

20. Is there anything else you'd like to share, recommend, or ask about your experience with being a Deep End Canada member that you haven't already shared so far?
